# Clinical characteristics and outcomes of lean diabetes mellitus in patients with moderate and severe aortic stenosis

**DOI:** 10.3389/fcvm.2026.1767022

**Published:** 2026-04-23

**Authors:** Aloysius Sheng-Ting Leow, Joy Yi-Shan Ong, Elizabeth Hui-En Thong, Andre Wen-Jie Seah, Aaron Kwun-Hang Ho, Elinor Yin-Lin Tan, Swee-Chye Quek, William Kok-Fai Kong, Tiong-Cheng Yeo, Ching-Hui Sia, Kian-Keong Poh

**Affiliations:** 1 Department of Cardiology, National University Heart Centre, Singapore; 2 Department of Medicine, National University Hospital, Singapore; 3 Department of Medicine, Yong Loo Lin School of Medicine, National University of Singapore, Singapore

**Keywords:** aortic stenosis, cardiometabolic risk, lean diabetes mellitus, major adverse cardiovascular events, metabolic syndrome, obesity paradox

## Abstract

**Background:**

Lean diabetes mellitus (DM), defined as the presence of DM in patients with low-normal body mass index (BMI), is an emerging concept associated with poorer cardiovascular and all-cause outcomes. We aimed to describe the clinical characteristics of aortic stenosis (AS) patients with concomitant lean DM, an understudied metabolic profile, and to evaluate its clinical relevance.

**Methods:**

In this retrospective cohort study involving 315 moderate and severe AS patients, identified from an echocardiographic database at a tertiary medical centre between September 2011 and December 2015, patients were stratified based on obesity (BMI ≥ or <23 kg/m^2^) and DM status. Outcomes were analysed over a follow-up period of 3.0 ± 2.1 years.

**Results:**

The mean age was 72.2 ± 13.1 years old, and 52.4% were female. Among the 315 patients with moderate and severe AS, 47 (14.9%) were lean DM, 24.4% were lean without DM, 30.0% were non-lean with DM, and 33.7% were non-lean without DM. Compared with non-lean DM patients, AS patients with lean DM were older (*p* < 0.001), and had a greater burden of cardiovascular co-morbidities, such as hypertension (*p* < 0.001) and coronary artery disease (*p* < 0.001). Lean DM was associated with an increased hazard of major adverse cardiovascular events (MACEs) on multivariable analysis (adjusted hazard ratio 1.81, 95% confidence interval 1.01–3.27, *p* = 0.046), after adjusting for demographics, co-morbidities, left ventricular ejection fraction, and AS severity.

**Conclusion:**

The presence of lean DM in moderate and severe AS patients portends a higher risk of MACE, and represents a high-risk phenotype that may warrant closer surveillance and earlier intervention.

## Introduction

Aortic stenosis (AS) is one of the most prevalent valvular heart diseases worldwide and contributes significantly to loss of functional status, quality of life, and longevity (1). In particular, degenerative, calcific valvular AS is the most common cause of AS in developed countries, associated with calcification which is highly correlated with old age and other metabolic conditions such as diabetes mellitus (DM), hypertension, and hyperlipidaemia (2). Compounded by the aging global population and increasing burden of metabolic disorders, the prevalence of AS has remained high and continues to increase, translating to rising mortality over the past 20 years (3, 4). Similar to AS, DM is a progressive condition and can influence the natural history of AS through various potential mechanisms, such as the acceleration of atherosclerosis or provoking a pro-inflammatory response to hyperglycaemic state, which results in a greater rate of degeneration of the aortic valve (AV) (5–7). In addition to the increased rate of AS progression, there is also evidence that DM has prognostic implications in AS patients, being associated with higher mortality in severe AS patients, and thus remains a clinically important co-morbidity to consider in the management of AS (8, 9).

DM and obesity are frequently observed together as common components of metabolic syndrome, leading to cardiovascular (CV) complications (10, 11). While traditionally thought to co-exist, the concept of lean DM as a unique entity has started to emerge, contrasting sharply with the current obesity epidemic (12). Defined as the presence of DM in patients with low-normal body mass index (BMI), lean DM has become better characterised by recent studies, which found that these patients are often male, have a history of childhood malnutrition, poor socioeconomic status, and relative early age of onset of DM (12). Observational studies in type 2 DM cohorts also suggest that lean DM patients often have poorer outcomes, with increased total, cardiovascular, and non-cardiovascular mortality when compared with obese DM patients (13, 14).

As part of metabolic syndrome, the confluence of obesity and DM—resulting in degenerative AS progression and poorer outcomes via accelerated atherosclerosis and heightened inflammation—appears intuitive. In contrast, lean DM is an understudied entity and its potential impact on valvular heart diseases, in particular AS, is poorly understood. Hence, we aimed to describe the clinical characteristics of AS patients with concomitant lean DM and to evaluate its prognostic impact.

## Methods

### Study design and population

**Figure 1:**
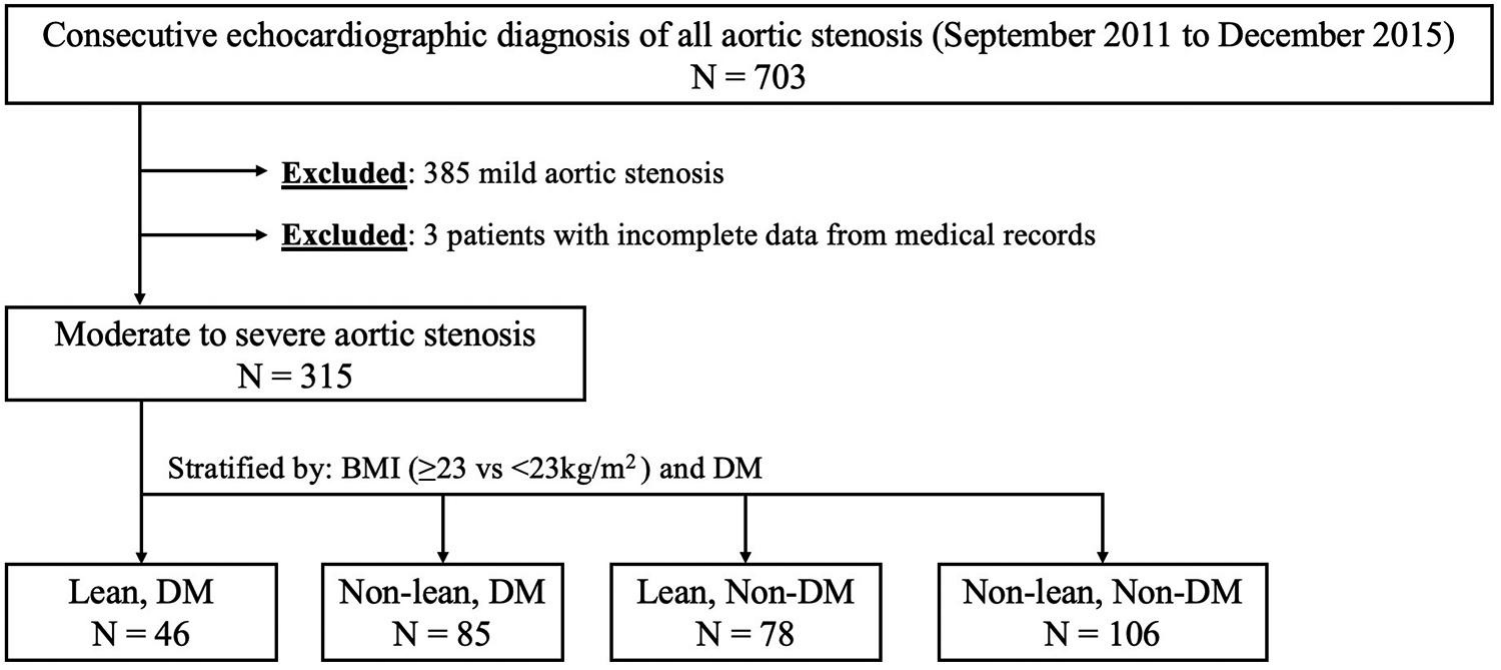
Patient selection flowchart. BMI, body mass index; DM, diabetes mellitus.

In this retrospective cohort study from a single tertiary medical centre, we first included all consecutive patients diagnosed with AS on echocardiography between September 2011 and December 2015. Ethics approval was obtained from the local institutional review board (2016/00358), and the requirement for informing consent was waived due to the retrospective nature of the study. For patients with multiple echocardiographic studies during the study period, only the index echocardiography was considered for the diagnosis of AS. The diagnosis and grading of AS severity (moderate and severe) were done in accordance with the guidelines of the European Association of Cardiovascular Imaging and the American Society of Echocardiography (15, 16). From a total of 703 consecutive AS patients diagnosed on echocardiography during the study period, we included 315 patients with moderate and severe AS, after excluding 385 patients with mild AS. Patients were then stratified into four groups based on their obesity and DM status at the time of AS diagnosis (Figure 1). BMI status was categorised based on a BMI of more than or equal to 23.0 kg/m^2^ (non-lean) vs. less than 23.0 kg/m^2^ (lean). A lower threshold of 23.0 kg/m^2^ was selected as it was recognised that the risks of cardiovascular disease and DM were increased at lower BMI points for Asian populations (17). DM was defined by documented clinician diagnosis in the electronic medical records (EMR) in accordance with the American Diabetes Association (ADA) diagnostic criteria (HbA1c ≥ 6.5%, fasting plasma glucose ≥7.0 mmol/L, plasma glucose ≥11.1 mmol/L during oral glucose tolerance test, or random plasma glucose ≥11.1 mmol/L accompanied by classic hyperglycaemic symptoms), and/or use of glucose-lowering therapy (18). Relevant data such as baseline demographics, clinical characteristics, echocardiographic parameters, treatments, and outcomes were then collected from the EMR and analysed.

### Study endpoints and statistical analysis

The primary endpoint studied was major adverse cardiovascular events (MACEs), a composite endpoint that included acute myocardial infarction (AMI), acute ischaemic stroke, and CV mortality. Secondary endpoints included hospitalisation due to CV conditions, heart failure (HF) hospitalisation, all-cause mortality, and AV replacement (AVR), which included both surgical and transcatheter AVR.

We presented categorical variables as frequencies and percentages, and continuous variables as mean ± standard deviations. The chi-square test (or Fisher’s exact test where appropriate) and the one-way analysis of variance (ANOVA) were used to analyse categorical and continuous variables, respectively. Survival curve analyses were performed using the cumulative incidence function estimate (to account for the competing risk of all-cause mortality) for the primary outcome of MACE and secondary outcomes of CV hospitalisation, HF hospitalisation, and AVR. Kaplan–Meier estimates were used for all-cause mortality, with the differences evaluated by log-rank tests (19). The multivariable regression models for the primary outcome of MACE and secondary outcomes of CV hospitalisation, HF hospitalisation, AVR, and all-cause mortality were adjusted for clinically relevant covariates selected *a priori* based on established literature, as opposed to univariate *p*-value filtering to avoid model instability and bias. Covariates included age (per year), sex, ethnicity, chronic kidney disease (CKD), previous AMI, left ventricular ejection fraction (LVEF) (per %), and AS severity. For heart failure hospitalisation, fewer covariates were adjusted for (age, female sex, ethnicity, LVEF, and AS severity) to minimise overfitting given the lower event rate. New York Heart Association (NYHA) class was not included in the multivariable models due to limited data availability and to minimise selection bias. Fine and Gray competing risks regression models were employed for the outcomes of MACE, CV hospitalisation, HF hospitalisation, and AVR (to account for all-cause mortality as a competing risk), while the Cox proportional hazards regression model was used for all-cause mortality. Results were presented as hazard ratios (HRs), 95% confidence intervals (95% CIs), and *p*-values (20). All *p*-values less than 0.05 were considered statistically significant. Competing risk analysis accounting for non-cardiovascular mortality was performed for the primary outcome of MACE. Exploratory subgroup analyses were also performed to evaluate for significant interaction between lean DM and age, sex for MACE outcome. Statistical analyses were performed using R Statistical Software (v4.4.1; R Core Team, 2023), Rstudio (v2024.9.0; Rstudio Team, 2024), with the following key packages: ggsurvfit (v1.0.0, Sjoberg, 2024) and tidycmprsk (v1.0.0, Sjoberg, 2023).

## Results

A total of 315 patients with at least moderate AS were included in this study, categorised into four groups: non-lean, non-DM (*n* = 106, 33.7%); non-lean, DM (*n* = 85, 30.0%); lean, non-DM (*n* = 77, 24.4%); and lean, DM (*n* = 47, 14.9%) (Table 1). The mean age of the cohort was 72.2 ± 13.1 years, with a balanced sex distribution of 52.4% women (*n* = 165). All diabetic patients had type 2 DM. Compared with non-lean DM patients, moderate and severe AS patients with lean DM were older (*p* < 0.001) and had greater cardiovascular co-morbidity burdens such as hypertension (*p* < 0.001) and coronary artery disease (*p* < 0.001). There were no significant differences in terms of AS severity (*p* = 0.112) and aetiology. Echocardiographic parameters are reported in Supplementary Table S1; notably, moderate and severe AS patients with concomitant lean DM had the smallest AV area (0.8 ± 0.3 cm^2^, *p* < 0.001), while the other echocardiographic parameters were similar across groups.

**Table 1:** Clinical characteristics of moderate and severe AS patients stratified by obesity and diabetes mellitus.

Variables	*N*	Overall	Lean, DM	Non-lean, DM	Lean, non-DM	Non-lean, non-DM	*p*-value
		*N* = 315	*N* = 46	*N* = 85	*N* = 78	*N* = 106	
Baseline Demographics							
Age (years), mean (SD)	315	72.2 (13.1)	76.7 (8.4)	71.0 (11.8)	73.8 (15.9)	70.1 (13.0)	**0**.**001**
Female sex, *n* (%)		165 (52.4)	23 (50.0)	44 (51.8)	40 (51.3)	58 (54.7)	0.943
Ethnicity, *n* (%)							**<0**.**001**
Chinese		197 (62.5)	34 (73.9)	49 (57.6)	61 (78.2)	53 (50.0)	
Malay		45 (14.3)	5 (10.9)	14 (16.5)	7 (9.0)	19 (17.9)	
Indian		22 (7.0)	4 (8.7)	6 (7.1)	0 (0.0)	12 (11.3)	
Others		51 (16.2)	3 (6.5)	16 (18.8)	10 (12.8)	22 (20.8)	
Height (cm), mean (SD)		158 (11)	158 (9)	158 (11)	157 (10)	157 (12)	0.941
Weight (kg), mean (SD)		63 (15)	52 (9)	72 (15)	51 (8)	69 (11)	**<0.001**
BSA (m^2^), mean (SD)		1.6 (0.2)	1.5 (0.2)	1.7 (0.2)	1.5 (0.2)	1.7 (0.2)	**<0.001**
BMI (kg/m^2^), mean (SD)		25.4 (5.9)	20.7 (2.1)	28.8 (5.7)	20.5 (2.1)	28.2 (5.3)	**<0.001**
Co-morbidities, *n* (%)							0.595
Current or ex-smoker		50 (15.9)	9 (19.6)	10 (11.8)	12 (15.4)	19 (17.9)	**<0.001**
Hypertension		224 (71.1)	43 (93.5)	69 (81.2)	42 (53.8)	70 (66.0)	**0.021**
Hyperlipidaemia		187 (59.4)	31 (67.4)	59 (69.4)	37 (47.4)	60 (56.6)	**<0.001**
Diabetes mellitus		131 (41.6)	46 (100.0)	85 (100.0)	0 (0.0)	0 (0.0)	**<0.001**
Coronary artery disease		121 (38.4)	28 (60.9)	39 (45.9)	19 (24.4)	35 (33.0)	0.061
Previous AMI		53 (16.8)	13 (28.3)	17 (20.0)	10 (12.8)	13 (12.3)	0.099
Known heart failure		49 (15.6)	11 (23.9)	13 (15.3)	15 (19.2)	10 (9.4)	0.660
Atrial fibrillation		52 (16.5)	9 (19.6)	11 (12.9)	12 (15.4)	20 (18.9)	0.700
Previous stroke or TIA		36 (11.4)	7 (15.2)	11 (12.9)	7 (9.0)	11 (10.4)	0.692
COPD		15 (4.8)	3 (6.5)	3 (3.5)	5 (6.4)	4 (3.8)	0.894
Malignancy		29 (9.2)	4 (8.7)	7 (8.2)	9 (11.5)	9 (8.5)	0.451
Chronic kidney disease		64 (20.3)	11 (23.9)	21 (24.7)	15 (19.2)	17 (16.0)	0.099
End-stage kidney disease		24 (7.6)	2 (4.3)	12 (14.1)	5 (6.4)	5 (4.7)	
HbA1c (%), mean (SD)	185	6.6 (1.6)	7.1 (1.5)	7.4 (1.9)	5.5 (0.5)	5.7 (0.6)	**<0**.**001**
NYHA class, *n* (%)	106						**<0**.**001**
Class 1		45 (42.5)	3 (20.0)	14 (43.8)	9 (45.0)	19 (48.7)	
Class 2		29 (27.4)	7 (46.7)	10 (31.3)	4 (20.0)	8 (20.5)	
Class 3		21 (19.8)	3 (20.0)	7 (21.9)	5 (25.0)	6 (15.4)	
Class 4		11 (10.4)	2 (13.3)	1 (3.1)	2 (10.0)	6 (15.4)	
AS characteristics							
AS severity, *n* (%)	315						0.115
Moderate		206 (65.4)	27 (58.7)	63 (74.1)	45 (57.7)	71 (67.0)	
Severe		109 (34.6)	19 (41.3)	22 (25.9)	33 (42.3)	35 (33.0)	
AS aetiology, *n* (%)	268						
Calcific degeneration		241 (89.9)	34 (87.2)	66 (90.4)	60 (93.8)	81 (88.0)	0.607
Bicuspid AV		26 (9.7)	3 (7.7)	6 (8.2)	6 (9.4)	11 (12.0)	0.883
Rheumatic		9 (3.4)	3 (7.7)	3 (4.1)	0 (0.0)	3 (3.3)	0.156
LVH, *n* (%)	312	208 (66.7)	31 (68.9)	53 (62.4)	54 (70.1)	70 (66.7)	0.746

AMI, acute myocardial infarction; AS, aortic stenosis; AV, aortic valve; BMI, body mass index; BSA, body surface area; COPD, chronic obstructive pulmonary disease; DM, diabetes mellitus; LVH, left ventricular hypertrophy; NYHA, New York Heart Association; SD, standard deviation; TIA, transient ischaemic attack.

Bold values indicate statistical significance, *p* < 0.05.

**Figure 2:**
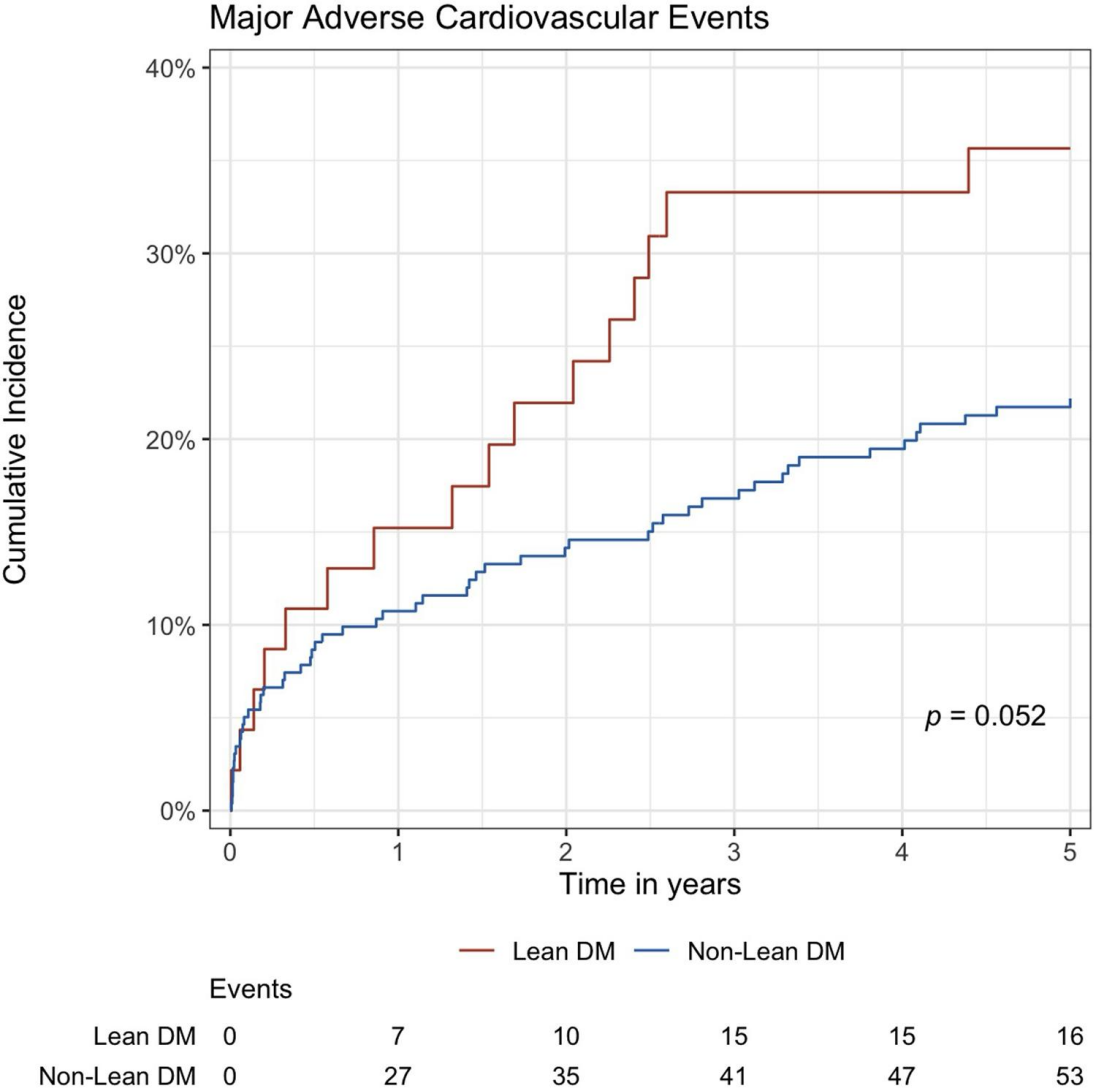
Cumulative incidence function estimate of major adverse cardiovascular events in moderate and severe AS patients stratified by presence of lean DM. AS, aortic stenosis; DM, diabetes mellitus.

Across a mean follow-up duration of 3.0 ± 2.1 years, there were no significant differences between the groups in terms of initial medical therapy, incidence of AVR (*p* = 0.618), MACE (*p* = 0.134), CV hospitalisation (*p* = 0.720), and HF hospitalisation (*p* = 0.530) (Table 2). Lean DM AS patients had significantly higher all-cause (*p* = 0.007) and CV mortality (*p* = 0.036) compared to other groups of moderate and severe AS patients. On survival curve analyses, lean DM AS patients had higher rates of MACE events than non-lean DM patients, but did not achieve statistical significance (*p* = 0.052) (Figure 2, Supplementary Figure S1). Rates of secondary outcomes (all-cause mortality, CV and HF hospitalisation, AVR) were also similar between lean DM and non-lean DM patients, as shown in Supplementary Figures S2A–D.

**Table 2:** Treatment and outcomes of moderate and severe AS patients stratified by obesity and diabetes mellitus.

Variables	*N*	Overall	Lean, DM	Non-lean, DM	Lean, non-DM	Non-lean, non-DM	*p*-value
		*N* = 315	*N* = 46	*N* = 85	*N* = 78	*N* = 106	
Treatment							
Medical therapy, *n* (%)	315						0.536
Aspirin		135 (42.9)	22 (47.8)	40 (47.1)	29 (37.2)	44 (41.5)	
Oral anticoagulation		41 (13.0)	5 (10.9)	10 (11.8)	11 (14.1)	15 (14.2)	0.917
Statin		185 (58.7)	27 (58.7)	56 (65.9)	39 (50.0)	63 (59.4)	0.234
ACEi or ARB		65 (20.6)	13 (28.3)	18 (21.2)	14 (17.9)	20 (18.9)	0.533
Beta blocker		121 (38.4)	15 (32.6)	34 (40.0)	26 (33.3)	46 (43.4)	0.439
Insulin		24 (7.6)	7 (15.2)	17 (20.0)	0 (0.0)	0 (0.0)	**<0** **.** **001**
OHGA		81 (25.7)	27 (58.7)	54 (63.5)	0 (0.0)	0 (0.0)	**<0** **.** **001**
AV replacement, *n* (%)		98 (31.1)	14 (30.4)	32 (37.6)	20 (25.6)	32 (30.2)	0.418
SAVR		60 (19.0)	11 (23.9)	17 (20.0)	13 (16.7)	19 (17.9)	0.770
TAVR		34 (10.8)	3 (6.5)	11 (12.9)	5 (6.4)	15 (14.2)	0.268
Concomitant CABG		16 (5.1)	0 (0.0)	9 (10.6)	3 (3.8)	4 (3.8)	0.055
Outcomes							
Follow-up duration (years), mean (SD)	315	3.0 (2.1)	2.9 (2.0)	3.1 (2.1)	2.7 (2.1)	3.3 (2.1)	0.328
AMI, *n* (%)		26 (8.3)	5 (10.9)	6 (7.1)	8 (10.3)	7 (6.6)	0.691
Acute ischaemic stroke, *n* (%)		9 (2.9)	1 (2.2)	3 (3.5)	3 (3.8)	2 (1.9)	0.872
CV hospitalisation, *n* (%)		70 (22.2)	13 (28.3)	20 (23.5)	16 (20.5)	21 (19.8)	0.672
HF hospitalisation, *n* (%)		35 (11.1)	4 (8.7)	10 (11.8)	6 (7.7)	15 (14.2)	0.527
All-cause mortality, *n* (%)		130 (41.3)	26 (56.5)	32 (37.6)	39 (50.0)	33 (31.1)	**0** **.** **008**
CV mortality, *n* (%)		47 (14.9)	13 (28.3)	14 (16.5)	9 (11.5)	11 (10.4)	**0** **.** **029**
MACE^a^, *n* (%)		69 (21.9)	16 (34.8)	18 (21.2)	17 (21.8)	18 (17.0)	0.112

ACEi, angiotensin-converting enzyme inhibitors; AMI, acute myocardial infarction; ARB, angiotensin receptor blockers; AS, aortic stenosis; AV, aortic valve; CABG, coronary artery bypass graft; CV, cardiovascular; DM, diabetes mellitus; HF, heart failure; MACE, major adverse cardiovascular events; OHGA, oral hypoglycaemic agents; SAVR, surgical aortic valve replacement; SD, standard deviation; TAVR, transcatheter aortic valve replacement.

a MACE included AMI, acute ischaemic stroke, and CV mortality.

Bold values indicate statistical significance, *p* < 0.05.

On regression analyses (Table 3), lean DM was associated with an increased hazard of MACE on univariate analysis (HR 1.75, 95% CI 1.01–3.05, *p* = 0.046). This association remained significant on multivariable analysis (aHR 1.81, 95% CI 1.01–3.27, *p* = 0.046) using the Fine and Gray competing risk model (accounting for all-cause mortality), after adjustments for age, sex, ethnicity, CKD, previous AMI, LVEF, and AS severity. Lean DM was not significantly associated with individual secondary outcomes (all-cause mortality, CV and HF hospitalisation, AVR) on both univariate and multivariable analyses. Exploratory subgroup analyses found that lean DM was associated with higher MACE incidence in older AS patients ≥75 years (HR 2.39, 95% CI 1.08–5.30, *p* = 0.032) and in male AS patients (HR 2.58, 95% CI 1.23–5.41, *p* = 0.012) (Supplementary Figure S3). However, the interaction term between lean DM and age or sex was not significant (*p* = 0.383 and *p* = 0.238 respectively).

**Table 3:** Association between lean diabetes and outcomes in moderate and severe AS patients.

Outcomes	Unadjusted HR (95% CI)	*p*-value	Adjusted HR^a^ (95% CI)	*p*-value
MACE^b^^,^^c^	1.75 (1.01–3.05)	**0**.**046**	1.81 (1.01–3.27)	**0**.**048**
All-cause mortality^b^	1.51 (0.98–2.33)	0.059	1.35 (0.87–2.10)	0.181
CV hospitalisation^b^	1.23 (0.68–2.21)	0.500	1.15 (0.62–2.14)	0.650
HF hospitalisation^d^	0.67 (0.24–1.88)	0.450	0.72 (0.24–2.15)	0.560
AV replacement^b^	0.84 (0.45–1.56)	0.590	0.94 (0.48–1.87)	0.870

AS, aortic stenosis; AV, aortic valve; CI, confidence interval; CV, cardiovascular; HF, heart failure; HR, hazard ratio; MACE, major adverse cardiovascular events.

a The Fine and Gray competing risks model (adjusting for all-cause mortality) was used for all outcomes except all-cause mortality; the Cox proportional hazards regression model was used for all-cause mortality.

b Adjusted for age, sex, ethnicity, chronic kidney disease, previous acute myocardial infarction, left ventricular ejection fraction, and AS severity.

c MACE included AMI, acute ischaemic stroke, and CV mortality.

d Adjusted for age, sex, ethnicity, left ventricular ejection fraction, and aortic stenosis severity.

Bold values indicate statistical significance, *p* < 0.05.

## Discussion

The key findings of this study are as follows: (1) moderate and severe AS patients with concomitant lean DM were older and had a higher burden of cardiovascular co-morbidities than their non-lean DM counterparts and (2) the presence of lean DM was independently associated with higher incidence of MACE in moderate and severe AS patients.

The association between aortic stenosis and traditional cardiovascular risk factors such as DM, hypertension, dyslipidaemia, and obesity is well established in the existing literature. Studies have found that these cardiovascular risk factors have an independent and direct relationship with incident AS (21, 22), with its development likely a result of the calcific degenerative process (23, 24). For example, exposure to high low-density lipoprotein (LDL)-cholesterol was found to increase the risk of symptomatic AS in a large cohort study (25). A previous study by Conrotto et al. had also reported that insulin-treated DM was independently associated with death and myocardial infarction in patients who underwent transcatheter aortic valve replacement (TAVR) (26). However, data on lean DM and its effects on cardiovascular conditions and outcomes in the aortic stenosis cohort remain limited (12, 13). Results from our study suggest that patients with lean DM represent a distinct high-risk subgroup among those with moderate and severe AS. These patients are generally older and possess higher cardiovascular disease burden such as coronary artery disease, previous strokes, and chronic kidney disease. In addition, while there was no significant difference in the severity of AS across groups, lean DM patients were observed to have a smaller aortic valve area, which suggests that advanced cardiac remodelling and aortic valve degeneration may occur earlier and more aggressively in AS patients with lean DM, possibly due to the combined metabolic and inflammatory effects, even in the absence of obesity (27).

There has been emerging data to suggest that lean DM is a clinically relevant and independent risk factor for adverse cardiovascular outcomes and all-cause mortality, but it remains an under-recognised metabolic phenotype especially in the context of valvular heart diseases (13, 14). While obesity has paradoxically been associated with improved outcomes in AS and TAVR cohorts—a phenomenon referred to as the “obesity paradox”—the impact of this phenomenon in DM patients has yet to be closely examined in these populations, and may yield unexpected results as DM and obesity are often seen in tandem (28–30). In our cohort, a significantly higher incidence of MACE was observed in AS patients with lean DM compared to non-lean DM patients after adjusting for relevant co-morbidities, which may suggest that the negative prognostic impact of lean DM may extend to AS patients as well. Several potential pathophysiological mechanisms may underlie this finding. Lean individuals with DM often exhibit higher levels of sarcopenia and frailty—possessing lower insulin-sensitive muscle mass which can contribute to the development of lean DM—and have been associated with increased cardiovascular risk and mortality due to poorer physiological reserves (31, 32). Furthermore, prior studies have reported higher high-sensitivity C-reactive protein levels in lean DM, suggesting underlying chronic low-grade inflammation that may contribute to endothelial dysfunction and atherosclerotic progression, thereby worsening cardiovascular outcomes (27, 33). The combined effects of these pathways were hypothesised to have led to the greater MACE risk observed in AS patients, suggesting the need for targeted risk stratification and management strategies in this high-risk subgroup.

Current guidelines for AS recommend definitive intervention, such as surgical or transcatheter AVR, primarily based on symptom severity and echocardiographic parameters such as valve gradient and aortic valve area (34, 35). However, given the higher risk of cardiovascular and all-cause mortality in this increasingly prevalent group of AS patients with lean DM—who may not necessarily meet the traditional criteria for intervention—clinicians should consider adopting a more personalised approach to risk stratify these patients (36). This would include the addition of muscle mass or body composition, inflammatory markers, and metabolic control in the targeted evaluation of lean DM patients. Beyond targeting improvement in valve haemodynamic via AVR, an integrated approach focusing on weight management, nutritional support, and intensive glycaemic control may also help mitigate the elevated cardiovascular risk in this vulnerable subgroup (37, 38). Lastly, AS patients with the high-risk phenotype of lean DM may require enhanced risk stratification and closer monitoring, given the poorer clinical trajectory observed.

### Strengths and limitations

This study is among the first to evaluate the impact of lean DM in AS patients, offering new insights into this group of high-risk patients beyond traditional cardiovascular risk factors (39). Furthermore, our study included a unique multi-ethnic Asian cohort, which is often under-represented in the existing literature largely dominated by Caucasian populations. This helps to broaden the applicability of risk models to non-Caucasian AS patients, particularly in the context of rising metabolic disorders in the Asia Pacific region (40). In addition, outcome analyses utilised the Fine and Gray competing risk regression models, which reduced survivor bias compared with the traditional Cox proportional hazards regression model, enhancing the validity of our findings. However, it is necessary to acknowledge several limitations inherent in this study. First, this was a retrospective, observational study, whereby unmeasured confounding factors, such as lipid parameters, may still be present despite adjustments. Therefore, we could only observe correlations and not draw causations. Second, as the study was conducted at a single Asian centre, the results may not be generalisable to other populations due to potential differences in body composition and DM phenotypes. Third, only the index echocardiography was used in the assessment of AS severity and LVEF, limiting the ability to account for subsequent changes in LVEF or progression of AS which could have affected outcomes observed during the study period. Fourth, there may be misclassification bias as the adjudication of obesity and DM status was based on a single time point during AS diagnosis. These conditions are dynamic and modifiable, and temporal fluctuations in BMI or glycaemic control, duration, and severity of DM could impact long-term prognosis. Therefore, it is challenging to fully and accurately capture the chronic burden of metabolic derangement on outcomes. There may also be potential misclassification bias as a lower BMI threshold of 23.0 kg/m^2^ was selected for our Asian population in contrast to the World Health Organisation (WHO) BMI cut-off of 25.0 kg/m^2^ for overweight. Finally, the size of the lean DM subgroup of AS patients was relatively small, limiting statistical power, particularly for secondary outcomes such as all-cause mortality and HF hospitalisation. Given the small sample size and modest number of MACE events observed, the findings should be taken as hypothesis-generating, necessitating large prospective future studies.

## Conclusion

In this cohort of moderate and severe AS patients, the presence of lean DM was independently associated with a higher incidence of MACE. These findings highlight lean DM as a high-risk phenotype in AS patients, underscoring the need for closer surveillance and tailored management.

## Data Availability

The raw data supporting the conclusions of this article will be made available by the authors without undue reservation.
