# Supplementary material for: Clinical characteristics and outcomes of lean diabetes mellitus in patients with moderate and severe aortic stenosis
Source: Front Cardiovasc Med. 2026 Apr 23;13:1767022. doi: 10.3389/fcvm.2026.1767022 (PMC13149384; doi:10.3389/fcvm.2026.1767022)

Supplementary Table 1. Echocardiographic parameters of moderate and severe AS patients stratified by obesity and diabetes mellitus

| **Variables** | **Overall** N = 315 | **Lean, DM** N = 46 | **Non-lean, DM** N = 85 | **Lean, Non-DM** N = 78 | **Non-lean, Non-DM** N = 106 | ***p*-value** |
| --- | --- | --- | --- | --- | --- | --- |
| AVA (cm^2^), mean (SD) | 0.9 (0.3) | 0.8 (0.3) | 1.0 (0.3) | 0.8 (0.3) | 1.0 (0.3) | **<0.001** |
| AVA index (cm^2^/m^2^), mean (SD) | 0.6 (0.2) | 0.6 (0.2) | 0.6 (0.2) | 0.5 (0.2) | 0.6 (0.2) | 0.314 |
| MPG (mmHg), mean (SD) | 37.4 (16.9) | 40.8 (18.8) | 36.1 (15.7) | 39.0 (18.1) | 35.9 (16.0) | 0.319 |
| Vmax (m/sec), mean (SD) | 372.5 (81.4) | 362.7 (83.1) | 370.1 (85.1) | 391.1 (74.6) | 364.8 (81.5) | 0.120 |
| LVEF (%), mean (SD) | 56.6 (14.1) | 56.6 (14.4) | 56.7 (13.7) | 53.7 (14.2) | 58.7 (13.9) | 0.136 |
| PASP (mmHg), mean (SD) | 38.4 (15.6) | 40.3 (20.0) | 37.7 (13.6) | 38.5 (16.4) | 38.1 (14.5) | 0.901 |
| LV mass index (g/m^2^), mean (SD) | 129.4 (41.7) | 127.4 (35.3) | 129.3 (41.6) | 133.4 (48.9) | 127.4 (38.8) | 0.826 |
| RWT, mean (SD) | 0.3 (0.2) | 0.3 (0.2) | 0.4 (0.2) | 0.3 (0.2) | 0.4 (0.2) | 0.090 |
| LVIDd (mm), mean (SD) | 48.6 (7.6) | 46.7 (7.5) | 50.0 (7.8) | 47.6 (7.2) | 49.0 (7.5) | 0.068 |
| LVIDs (mm), mean (SD) | 32.4 (8.8) | 31.9 (8.5) | 33.8 (9.6) | 31.6 (8.1) | 32.2 (8.9) | 0.441 |
| IVSd (mm), mean (SD) | 10.8 (2.5) | 10.8 (2.7) | 11.0 (2.8) | 10.9 (2.0) | 10.7 (2.5) | 0.937 |
| IVSs (mm), mean (SD) | 15.4 (3.1) | 14.8 (2.7) | 15.6 (3.5) | 14.9 (2.8) | 15.8 (3.1) | 0.082 |
| LVPWd (mm), mean (SD) | 11.1 (2.0) | 11.2 (2.1) | 11.4 (2.2) | 10.9 (2.1) | 11.1 (1.7) | 0.562 |
| LVPWs (mm), mean (SD) | 15.4 (2.7) | 15.5 (3.0) | 15.6 (2.8) | 15.2 (2.5) | 15.3 (2.7) | 0.733 |
| LVEDV (ml), mean (SD) | 114.5 (42.7) | 104.7 (39.8) | 122.3 (46.1) | 108.9 (38.6) | 116.6 (43.1) | 0.080 |
| LVEDV index (ml/m^2^), mean (SD) | 69.8 (25.4) | 69.6 (25.0) | 69.4 (25.9) | 73.7 (26.8) | 67.3 (24.0) | 0.436 |
| LVESV (ml), mean (SD) | 47.4 (34.9) | 45.2 (30.0) | 52.6 (38.8) | 44.0 (28.8) | 46.6 (37.5) | 0.433 |
| LVESV index (ml/m^2^), mean (SD) | 28.9 (20.7) | 29.0 (18.4) | 30.6 (22.9) | 29.2 (19.0) | 27.3 (21.0) | 0.780 |

Abbreviations: AS – aortic stenosis; AVA – aortic valve area; DM – diabetes mellitus; IVS – interventricular septum; LV – left ventricular; LVEDV – left ventricular end diastolic volume; LVEF – left ventricular ejection fraction; LVESV – left ventricular end systolic volume; LVID – left ventricular internal diameter; LVPW – left ventricular posterior wall diameter; MPG – mean pressure gradient; PASP – pulmonary artery systolic pressure; RWT – relative wall thickness; SD – standard deviation; Vmax – peak aortic jet velocity

Supplementary Figure 1. Cumulative incidence function estimate of major adverse cardiovascular events in moderate and severe AS patients stratified by obesity and diabetes mellitus status


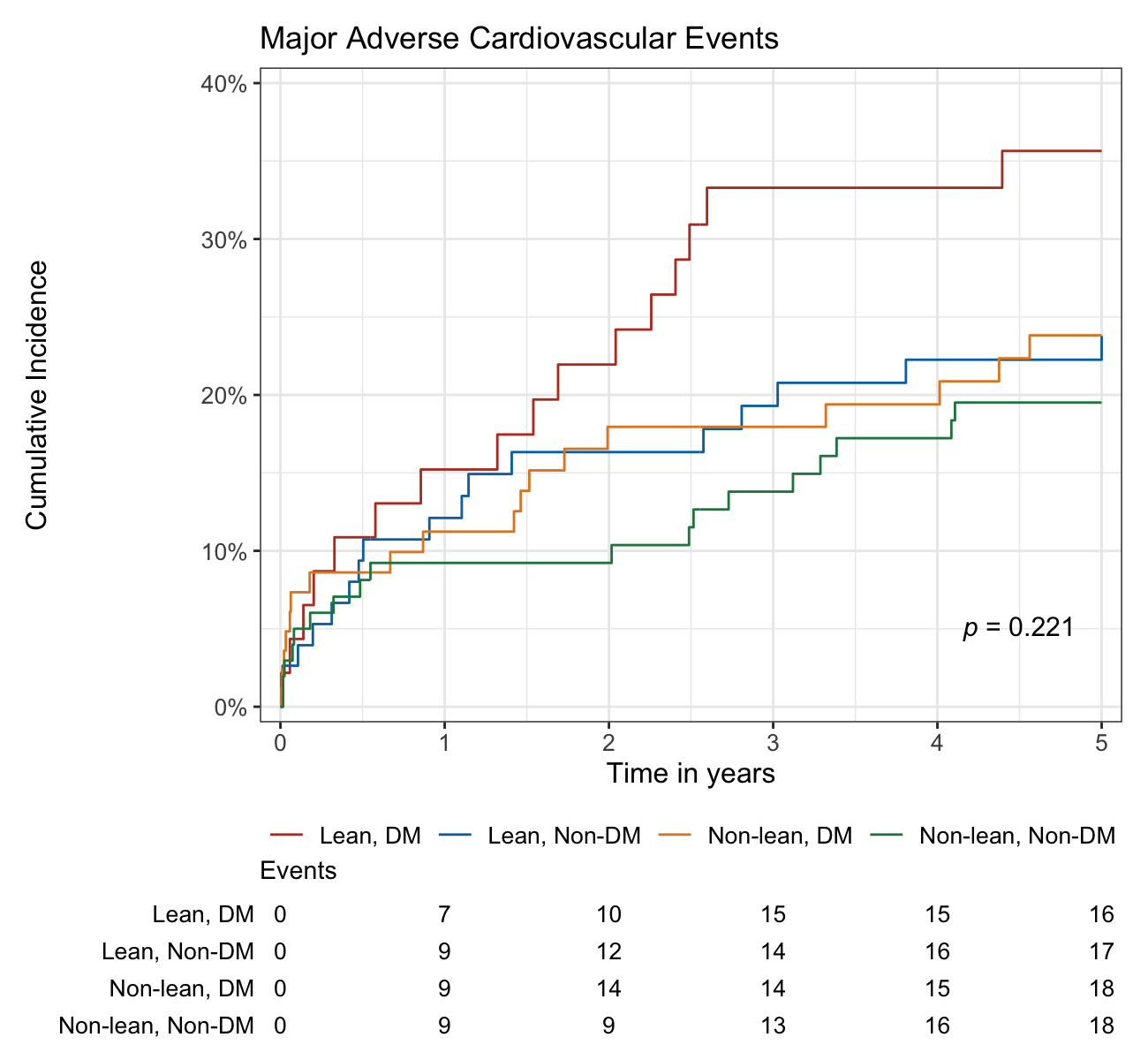


Supplementary Figure 2(A-D). Cumulative Incidence Function estimates of outcomes in moderate and severe AS patients stratified by presence of lean DM


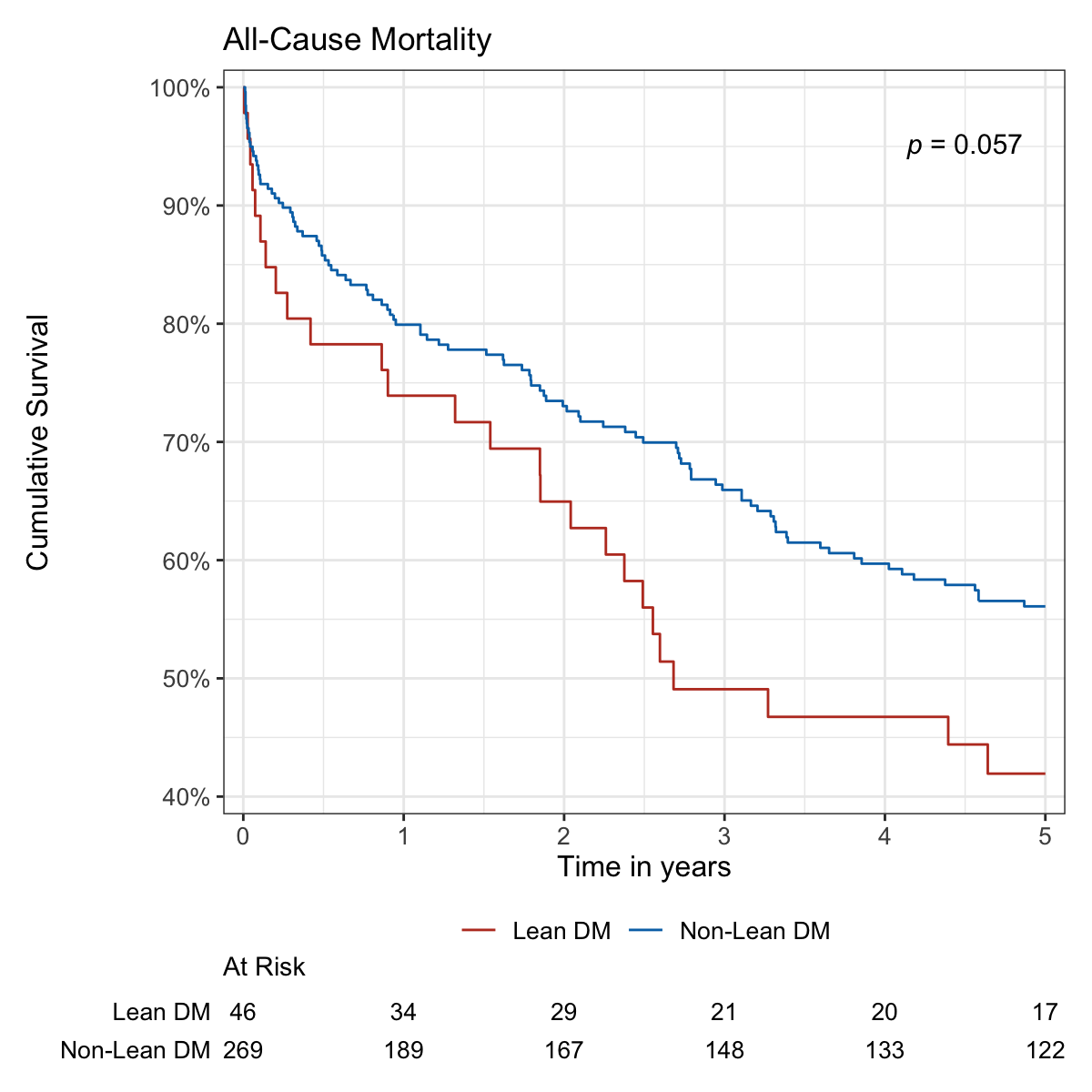

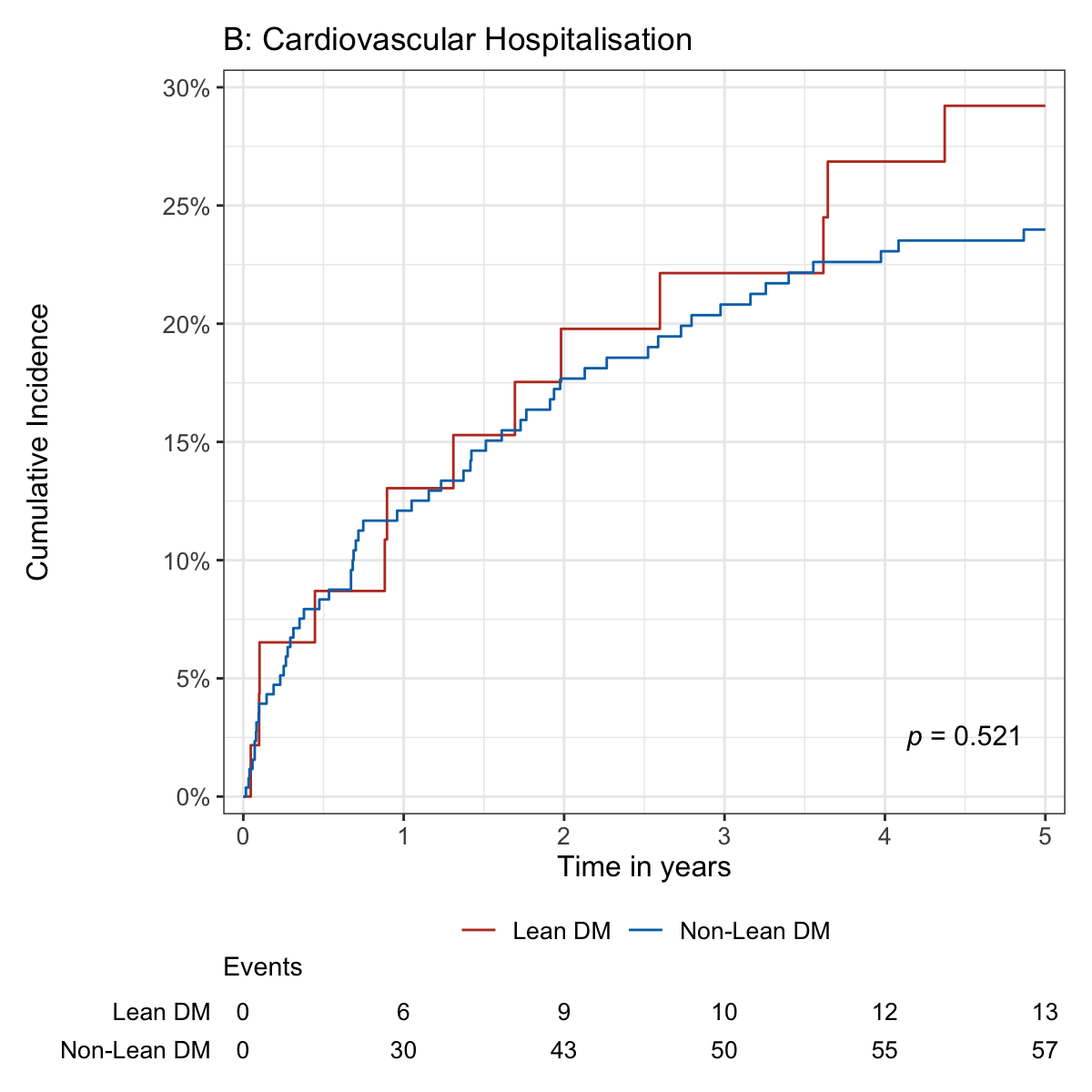


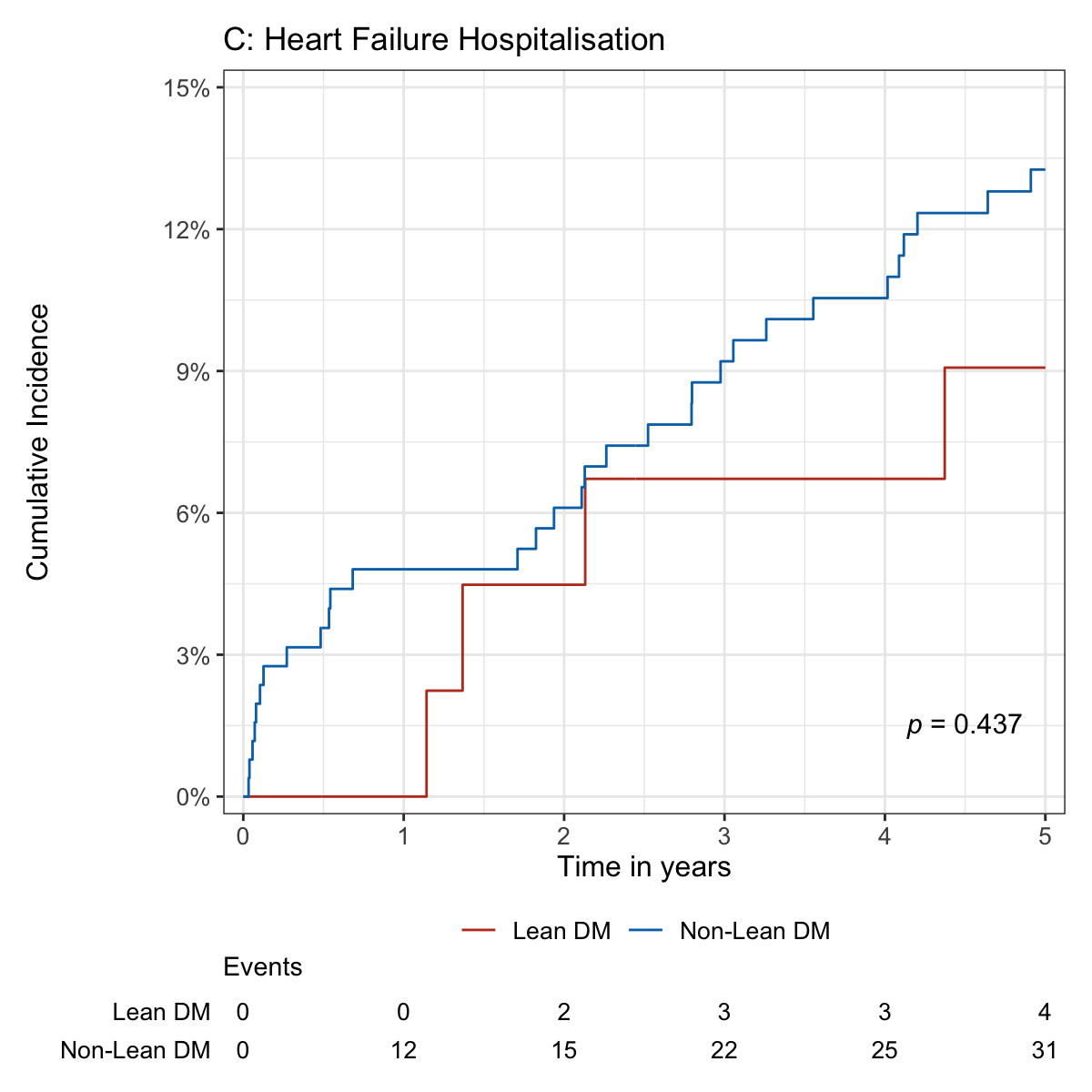

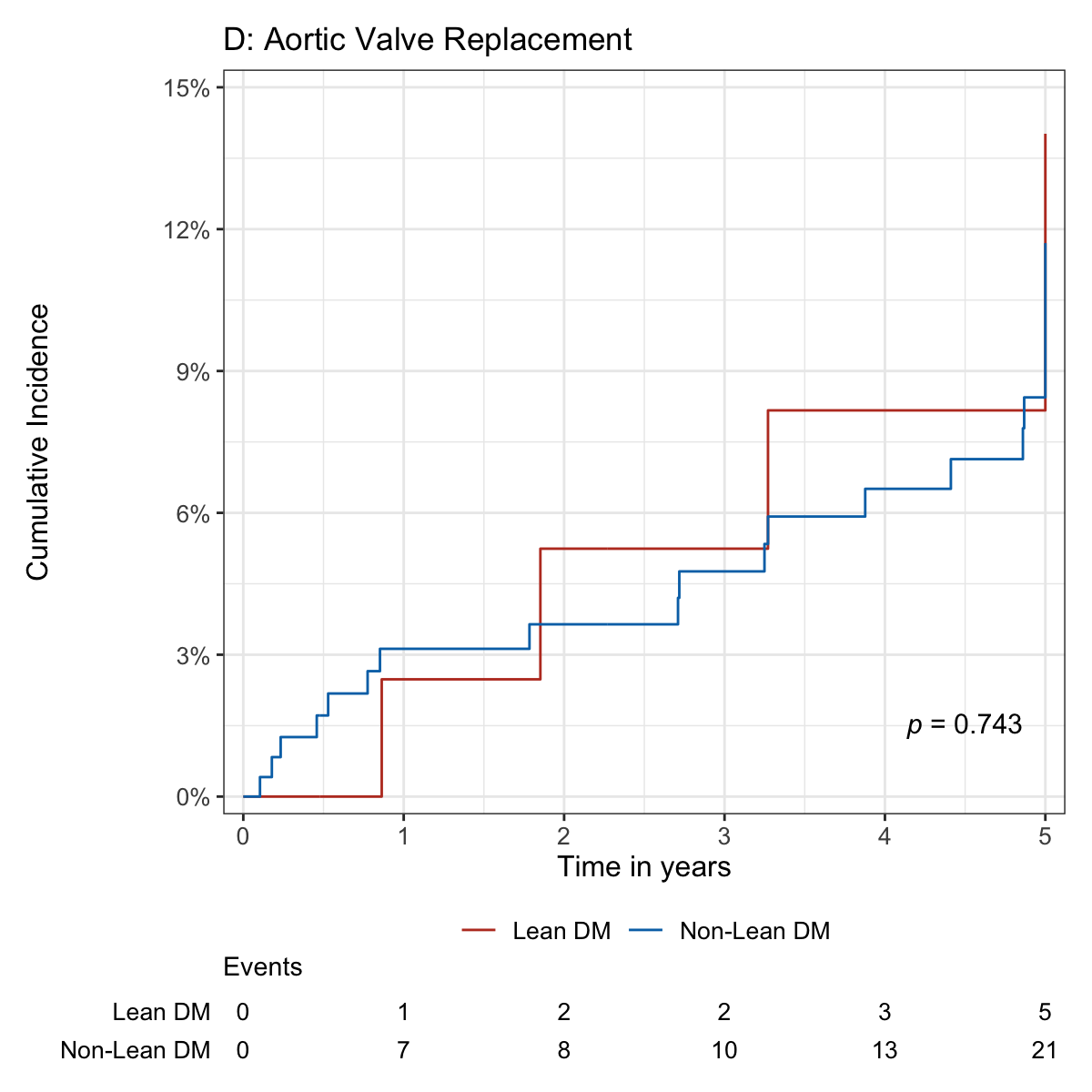


Abbreviations: AS – aortic stenosis; DM – diabetes mellitus

Supplementary Figure 3. Forest plot of subgroup interactions of MACE outcomes in moderate and severe AS patients


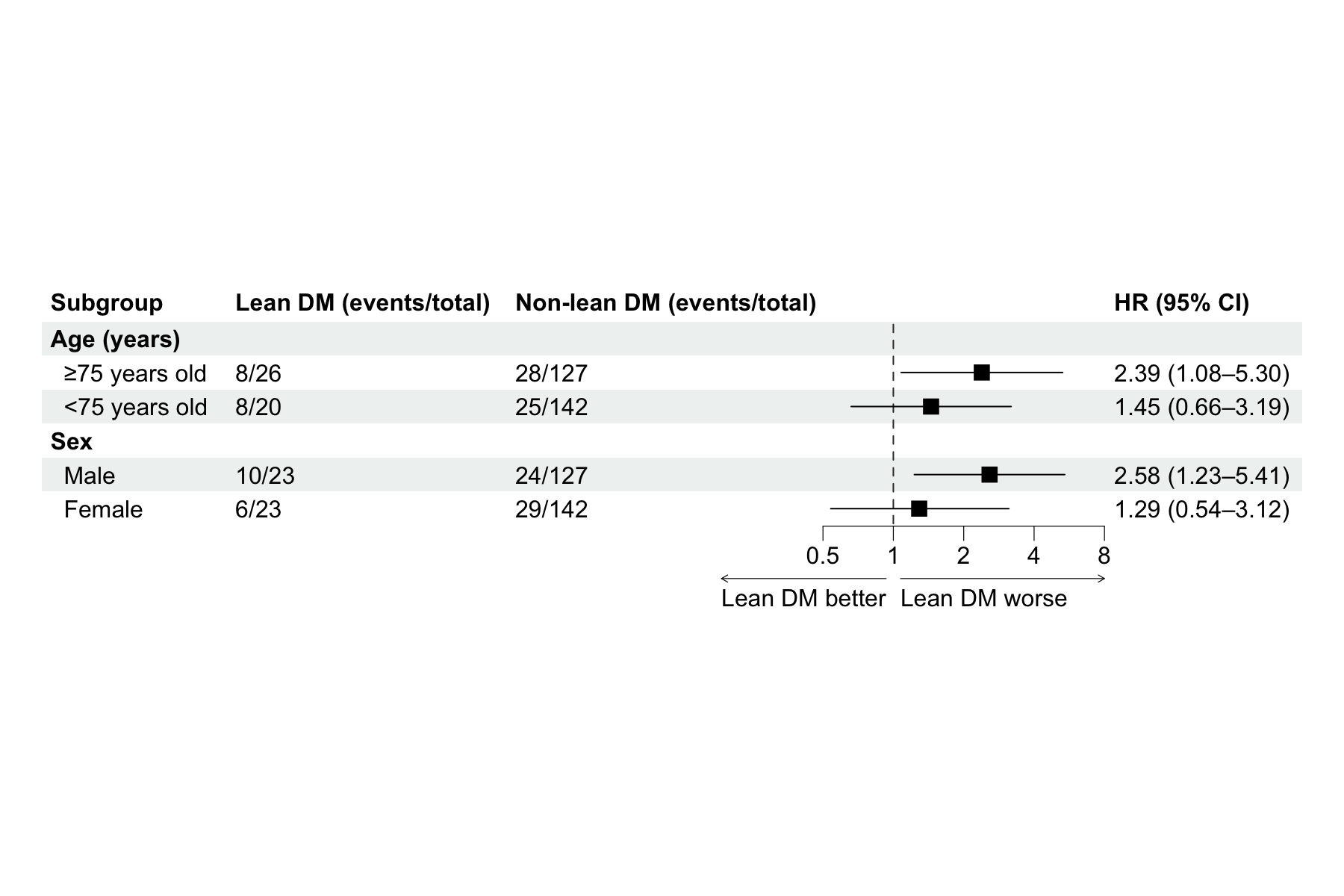

Supplement: Supplementary file 1 [file Datasheet1.docx]
